# Influence of early antioxidant supplements on clinical evolution and organ function in critically ill cardiac surgery, major trauma, and subarachnoid hemorrhage patients

**DOI:** 10.1186/cc6981

**Published:** 2008-08-07

**Authors:** Mette M Berger, Ludivine Soguel, Alan Shenkin, Jean-Pierre Revelly, Christophe Pinget, Malcolm Baines, René L Chioléro

**Affiliations:** 1 Department of Intensive Care Medicine & Burns Centre, University Hospital (Centre Hospitalier Universitaire Vaudois, CHUV), Rue du Bugnon 46, CH-1011 Lausanne, Switzerland; 2 Department of Clinical Chemistry, Royal Liverpool University Hospital and University of Liverpool, Liverpool, UK; 3 Health Technology Assessment Unit, CHUV, Rue du Bugnon 46, CH-1011 Lausanne, Switzerland

## Abstract

**Introduction:**

Oxidative stress is involved in the development of secondary tissue damage and organ failure. Micronutrients contributing to the antioxidant (AOX) defense exhibit low plasma levels during critical illness. The aim of this study was to investigate the impact of early AOX micronutrients on clinical outcome in intensive care unit (ICU) patients with conditions characterized by oxidative stress.

**Methods:**

We conducted a prospective, randomized, double-blind, placebo-controlled, single-center trial in patients admitted to a university hospital ICU with organ failure after complicated cardiac surgery, major trauma, or subarachnoid hemorrhage. Stratification by diagnosis was performed before randomization. The intervention was intravenous supplements for 5 days (selenium 270 μg, zinc 30 mg, vitamin C 1.1 g, and vitamin B_1 _100 mg) with a double-loading dose on days 1 and 2 or placebo.

**Results:**

Two hundred patients were included (102 AOX and 98 placebo). While age and gender did not differ, brain injury was more severe in the AOX trauma group (*P *= 0.019). Organ function endpoints did not differ: incidence of acute kidney failure and sequential organ failure assessment score decrease were similar (-3.2 ± 3.2 versus -4.2 ± 2.3 over the course of 5 days). Plasma concentrations of selenium, zinc, and glutathione peroxidase, low on admission, increased significantly to within normal values in the AOX group. C-reactive protein decreased faster in the AOX group (*P *= 0.039). Infectious complications did not differ. Length of hospital stay did not differ (16.5 versus 20 days), being shorter only in surviving AOX trauma patients (-10 days; *P *= 0.045).

**Conclusion:**

The AOX intervention did not reduce early organ dysfunction but significantly reduced the inflammatory response in cardiac surgery and trauma patients, which may prove beneficial in conditions with an intense inflammation.

**Trials Registration:**

Clinical Trials.gov RCT Register: NCT00515736.

## Introduction

Critically ill patients are generally exposed to an increased oxidative stress, which is proportional to the severity of their condition [1,2]. A network of functionally overlapping antioxidant (AOX) defense mechanisms aims at protecting cells from reactive oxygen and nitric oxide species. It is formed by trace-element-dependent enzymes such as superoxide dismutase, catalase, and glutathione peroxidase (GPX) (selenium, zinc, manganese, copper, and iron), thiol donors, and their precursors. The vitamins E, C, and β-carotene with other molecules (urate and albumin) also contribute to AOX defense [3]. The more severe the insult and the sepsis, the larger the depletion of AOXs appears to be [1,4,5]. The micronutrients have the capacity to downregulate nuclear factor-kappa-B activation and subsequent cytokine production [6]. Hence, micronutrient deficiency favors the persistence of inflammation and the propagation of lipid peroxidation and other free-radical-mediated damage, contributing to organ dysfunction and failure.

The selenium status of the general population in Europe is often suboptimal before acute illness [7,8]. Nearly all patients with sepsis or shock exhibit early low plasma selenium levels, which are correlated with the severity of inflammation and subsequent outcome [9]. Micronutrient status deteriorates during acute illness despite standard micronutrient intake [10]. Some therapies, such as continuous renal replacement, increase micronutrient losses and may further reduce micronutrient availability [11]. In addition, the acute-phase response causes redistribution of micronutrients from the vascular compartment to the liver and the reticuloendothelial system, depleting the circulating micronutrients [12].

Much clinical research has focused on the AOX micronutrients vitamins C and E, copper, selenium, and zinc. Most clinical trials have been carried out in sepsis, trauma, and burns, which are characterized by intense oxidative stress and inflammatory response. In a systematic review of randomized studies [13], overall AOX supplements were associated with a significant reduction in mortality (relative risk [RR] 0.65, 95% confidence interval [CI] 0.53 to 0.80; *P *< 0.0001) but had no effect on infectious complications. In further subgroup analyses, selenium supplementation was associated with a nonsignificant reduction in mortality (RR 0.59, 95% CI 0.32 to 1.08; *P *= 0.09). Recent studies of selenium, copper, and zinc supplements in burn patients [14] and high-dose selenium in severe sepsis [15] confirm these positive observations: the reinforcement of the AOX defenses is a plausible mechanism [3]. The population likely to benefit from such interventions has not been defined yet. While selenium supplementation, or substitution, is rational in the European selenium-depleted areas [7], the physiology of the endogenous AOX system should be considered. AOX defenses act as a network [16]. The AOXs therefore should probably be provided as a combination to avoid disequilibrium in the system, especially if several micronutrients are lost simultaneously as in trauma, burns, and renal replacement therapy.

The present trial aimed at testing the hypothesis that the early administration of an AOX micronutrient combination including selenium would improve clinical outcome in selected groups of critically ill patients admitted for conditions characterized by local or systemic oxidative stress [17,18] and at risk of micronutrient depletion, by reinforcing the endogenous AOX defenses and reducing organ failure.

## Materials and methods

### Study design

We conducted a prospective, randomized, double-blind, placebo-controlled, single-center trial with the approval of the institutional ethics committee.

### Patient population

Two hundred consecutive patients admitted to the intensive care unit (ICU) at the University Hospital (Centre Hospitalier Universitaire Vaudois) of Lausanne were enrolled from January 2003 through September 2004. Three conditions admitted with organ failure deemed likely by the medical team to require at least 48 hours of ICU treatment were considered: cardiac valve or coronary bypass surgery with postoperative cardiac or respiratory failure, major trauma (with or without brain injury) with an Injury Severity Score (ISS) of greater than 9, and severe subarachnoid hemorrhage (SAH) (that is, World Federation of Neurological Surgeons grades 3, 4, and 5) [19]. These three pathologies were investigated based on the demonstration of oxidative stress-related damage [17,18,20]. Exclusion criteria were absence of consent, participation in another study, liver cirrhosis or major burns, and life expectancy of less than 48 hours or a lack of commitment to full aggressive care (anticipated withholding or withdrawing of treatments in the 48 hours).

### Severity of condition

Cardiac surgery patients' preoperative status was assessed using the Parsonnet score [21] and the Euroscore [22]. Trauma severity was based on the ISS [23] and the Glasgow Coma Scale (GCS) score on admission and at discharge. Severity of physiological condition was determined by the first 24 hours' Simplified Acute Physiology Score (SAPS II) [24] and the daily sequential organ failure assessment (SOFA) [25] scores. Pre-existing renal failure was defined as a preadmission creatinine clearance of less than 60 mL/minute (measured or calculated with the Cockcroft-Gault equation on preoperative creatinine value [26]).

### Randomization

After stratification for diagnosis, the patients were randomly assigned by the pharmacist to either AOX micronutrient or placebo group, using a random list with a four-block allocation. Patients, clinicians, and investigators were blinded to the treatment. Black plastic bags covered the solutions, and colored tubing was used for infusion. Trace elements and vitamins were prepared in separate bags. Labels carried the patients' name, study number, and whether the content was supposed to be vitamin or trace element. The ethics committee, considering the limited risks associated with the micronutrient supplements, delivered a waiver of consent for the study enabling the randomization, the initiation of the intervention, and the first blood sampling. Oral consent was requested within 48 hours and a written consent within 96 hours. The patient was asked first whether he/she was not competent at that time, and provisory consent was requested among the relatives and confirmed by the patient once his/her condition had recovered.

### Intervention

Intervention consisted of delivering either AOX supplement or placebo for 5 days starting within 24 hours of admission. The supplementation consisted of an initial loading dose (double dose for 2 days) followed by 3 days of therapeutic dose (Table 1). The intervention solutions were infused alternately intravenously (iv) over the course of 10 to 12 hours each (vitamins during the daytime and trace elements over night). Products were Decan^®^, Sodium selenite^®^, and Zinc gluconate^® ^(Laboratoires Aguettant, Lyon, France), Soluvit^® ^+ Vitalipid^® ^(Fresenius Kabi AG, Bad Homburg, Germany), vitamin B_1 _as Benerva^® ^and vitamin C (Streuli Pharma AG, Uznach, Switzerland), and α-tocopherol as Ephynal^® ^(Roche, Basel, Switzerland). Vitamin E was delivered by nasoenteric tube. Besides the intervention solution, all patients received the ICU's 'standard vitamin profile' consisting of 100 mg thiamine and 500 mg vitamin C iv per day. In case of suspicion of alcohol abuse, an additional 100 mg thiamine was delivered. The 11 patients requiring parenteral nutrition (PN) for a total of 82 days (3 in the placebo group and 8 in the AOX group; *P *= 0.13) received the recommended doses of micronutrients for PN in addition to the 'intervention solution' (1 ampule of Soluvit^® ^+ 1 ampule of Vitalipid^® ^+ 1 ampule of Decan^®^) as part of standard care. In patients on full enteral nutrition (EN), one multivitamin and mineral tablet (Supradyn^®^; Roche) were delivered per day with EN (these micronutrients are not included in Table 1).

**Table 1:** Total antioxidant micronutrient doses in supplements during the first 5 days

Micronutrient	Days 1 and 2	Days 3–5
Zinc, mg	60	30
Selenium, μg	540.4	270.2
Vitamin C, mg	2,700^a^	1,600^a^
Vitamin B_1_, mg	305^a^	102.5
Vitamin E enteral, mg	600	300
Vitamin E iv, mg	12.8	6.4

^a^Includes the standard supplementation policy that was provided to both groups (500 mg vitamin C/day for 5 days and 100 mg vitamin B_1_/day for 3 days). iv, intravenously.

### Outcome variables

The primary outcome variable was a change in the acute kidney injury (AKI) score. Changes in organ function monitored by the SOFA score [25] were considered as an important secondary endpoint but were not used to calculate the sample size. At the time of the trial initiation, the SOFA scores' capacity to detect changes in mainly cardiac and trauma patients was not known. Renal failure affects about 35% of critically ill patients [27] and remains a major determinant of length of hospital stay [28]. Three levels of severity were considered: (a) AKI based on acute alterations of urine output according to the AKI Network [29] (stage 1: urine output of less than 0.5 mL/kg for 6 hours; stage 2: urine output of less than 0.5 mL/kg for 12 hours; and stage 3: urine output of less than 0.3 mL/kg for 12 hours or anuria for 12 hours). Acute renal failure was further defined by a plasma creatinine increase of (b) 50 or (c) 90 μmol/L [30]. The SOFA score was further used to test global organ dysfunction: this score ascribes a value of severity of organ failure from 0 to 4 for cardiovascular, respiratory, renal, hepatic, nervous, and coagulation failure (maximum score of 24). The SOFA score was repeated daily until day 5 or until discharge from the ICU. Secondary outcome variables included the daily worst arterial partial pressure of oxygen/fraction of inspired oxygen (PaO_2_/FiO_2_) ratio and mechanical ventilator dependence. The number of ventilator-free days to day 30 was defined as the number of days of unassisted breathing to day 30 after randomization, assuming a patient survives and remains free of invasive or noninvasive assisted breathing for at least 2 consecutive calendar days after extubation, whatever the vital status at day 30. Infectious complications, duration of ICU stay (counted by quarter-days rounded to the closest 6 hours), and ICU, hospital, and 3-month mortality rates were recorded. All infectious complications were recorded using the Centers for Disease Control and Prevention definitions [31], with special emphasis on pulmonary infections: pneumonia was defined as the combination of systemic inflammatory response syndrome (SIRS) with a new infiltrate on the chest x-ray (or progression of an infiltrate), a new or persistent hypoxemia, and purulent sputum. A standardized questionnaire was used to assess quality of life at 3 months: the Medical Outcome Study Short Form 36-item health survey (MOS SF-36). The patients were contacted by telephone, and the questionnaire was limited to the physical components: physical activity (physical functioning [PF]: scores 10 to 30), limitation due to physical status (role functioning-physical [RP]: scores 4 to 8), pain (bodily pain [BP]: scores 2 to 12), perceived health (general health perception [GH]: scores 5 to 25), and summary physical score of the MOS SF-36 were analyzed [32]. Due to interview difficulties, psychological components were not elicited.

### Laboratory determinations

Plasma creatinine, C-reactive protein (CRP), glucose, albumin, leukocytes, and platelets were determined daily for clinical purposes, and aspartate amino transferase, alanine amino transferase, and urate were determined three times weekly using standard clinical laboratory methods. Blood samples were collected on admission (day 0) and on day 5 (end of supplementation) to determine plasma zinc and selenium: analysis was in duplicate by inductively coupled plasma mass spectrometry (Plasmaquad 3 ICP-MS; VG Elemental, Winsford, Cheshire, UK) using aqueous inorganic standards. All plasma specimens were diluted in 1% nitric acid/0.2% n-butanol/0.2% n-propanol and 10 parts per billion indium as internal standard [33]. Plasma GPX was determined by the RANSEL method (Randox Laboratories, Belfast, UK).

### Nutritional support

EN or PN was initiated on a clinical basis, according to the unit's clinical protocols with the standard ICU solutions. The energy target was calculated as 1.2 times the predicted resting energy from the Harris and Benedict equation in cardiac surgery and SAH patients and as 30 kcal per kg body weight in trauma patients. EN was considered first, and PN was used only when EN was contraindicated. Glucose-insulin infusions were delivered in those at risk of ischemic postoperative cardiac failure. The cumulated energy balance was calculated at 5 days and at the end of the ICU stay. For those patients discharged before day 5, the energy intakes were recorded until day 5.

### Blood glucose control

The blood glucose target of 5 to 8 mmol/L was achieved by means of a continuous insulin infusion. For every patient, a mean of all blood glucose values per 24 hours was calculated during the first 5 days. The 24 hours' insulin doses were recorded. The variables were retrieved from the database of our clinical information system (MetaVision^®^; *i*MD *soft*, Tel Aviv, Israel).

### Statistical analysis

As there were no data available in the literature on the expected impact of supplementation on the SOFA score, the sample size was determined based on an expected reduction of acute renal failure of 20% defined as a creatinine increase of 50 μmol/L [34], an organ failure that has a significant impact on outcome. We realized an *a priori *power analysis, expecting an acute renal failure incidence of 30% and a 50% reduction, using an alpha level of 0.05 and a power of 0.9: these numbers resulted in a sample size of 186 (rounded to 200). A safety committee was formed to address safety issues, but it could not modify the sample size. After 60 and 120 patients, respectively, two meetings were conducted in order to detect over-mortality and adverse events: the two intermediate analyses did not detect any difference between groups.

Analysis was by intent to treat. Data are presented as mean ± standard deviation or as median and range when specified. Demographic data, energy balance, and baseline variables were analyzed by one-way analysis of variance (ANOVA) as they were normally distributed; two-way ANOVAs were used for variables repeated over time such as SOFA score, PaO_2_/FiO_2 _ratios, laboratory variables, and insulin dose. *Post hoc *comparisons were carried out by Dunnett test (effect of time versus baseline in each group) or Scheffe test (between-group comparisons at the same time point), where appropriate. Rank tests were used for nonparametric variables. Kaplan-Meier analysis was applied to length of hospital stay: nonsurvivors were considered as never achieving the event of interest (discharge) and censored at the end of evaluation period. Multiple and simple logistic regressions between variables were calculated. Significance was considered at a *P *value of less than 0.05; trends were considered up to a *P *value of less than 0.25. The statistical package was JMP^® ^version 5.1. (SAS Institute Inc., Cary, NC, USA).

## Results

**Figure 1:**
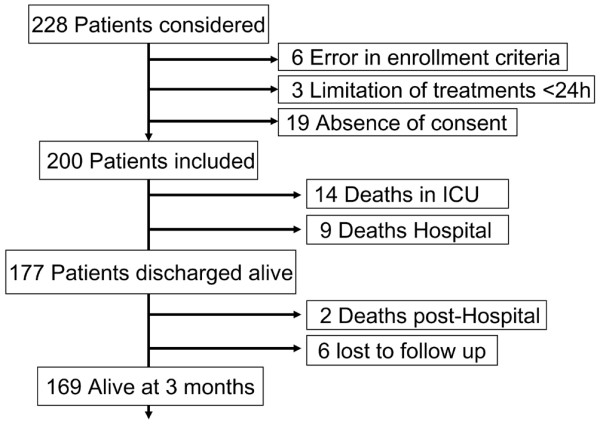
Enrollment diagram. ICU, intensive care unit.

Altogether, 200 patients completed the trial, resulting in 1,609 days of ICU treatment included in the analysis (Figure 1). A further 28 patients were considered but did not fulfill the study criteria, were deemed too severe within 24 hours with limitation of treatment, or refused consent. Table 2 shows the global patient characteristics and their distribution in the three diagnostic categories: age, gender ratio, SAPS II, body mass index, and SOFA score did not differ between the AOX and placebo groups, while the cardiac surgery patients were significantly older than both trauma and SAH groups. The gender ratio differed in trauma patients (predominance of males) and in the SAH group (predominance of females). The mean SAPS II was 38 ± 13 (predicted mortality 24.8%) and was highest in the cardiac surgery patients. Some heterogeneity was observed despite the randomization [35]. The cardiac scores did not differ significantly between groups: median Parsonnet was 15.5 (range 3 to 53) and median Euroscore was 8 (range 1 to 17). While severity of disease was similar in the cardiac and SAH treatment groups, there were significant differences regarding severity of brain injury between the trauma AOX and placebo patients (Table 3). Severity of brain injury was worse in the AOX subgroup compared with placebo patients, as reflected by a lower admission GCS score (of 8) (*P *= 0.11), with more severe SAPS (due to the low GCS score; *P *= 0.04) and brain ISS (*P *= 0.019) and worse admission neurological SOFA scores (*P *= 0.012). On discharge, GCS score differed significantly among brain-injured patients, with 11.1 ± 4.2 in the AOX group versus 13.6 ± 4.2 in the placebo group (*P *= 0.03), while there was no difference in the 27 patients without brain injury (15 ± 0 and 14.9 ± 0.3, respectively). These differences were associated with 6 versus 2 deaths in the placebo group (*P *= 0.16).

**Table 2:** Patient characteristics on admission with detail of the diagnostic categories

Variable	All		Complex cardiac surgery		Trauma		Subarachnoid hemorrhage	
Number	200		113		66		21	
	AOX	Placebo	AOX	Placebo	AOX	Placebo	AOX	Placebo
Number	102	98	57	56	34	32	11	10
Gender, male/female	127/73		70/43^a^		52/14^a^		5/16^a^	
Age, years	59 ± 19		70 ± 10^a^		40 ± 19^a^		54 ± 9	
	58 ± 19	59 ± 20	69 ± 8	71 ± 11	40 ± 19	40 ± 19	54 ± 7	53 ± 10
Weight, kg	74 ± 15		75 ± 16		75 ± 13		68 ± 12	
	74 ± 15	74 ± 15	75 ± 16	74 ± 17	72 ± 12	77 ± 13^b^	72 ± 13	64 ± 9^c^
Body mass index, kg/m^2^	25.8 ± 4.6		26.5 ± 5.0		24.9 ± 3.9		24.5 ± 3.4	
	25.8 ± 4.8	25.7 ± 4.3	26.7 ± 5.3	26.2 ± 4.7	24.2 ± 3.8	25.6 ± 3.9	25.2 ± 4.0	23.7 ± 2.5
SAPS II	37.5 ± 12.7		39.3 ± 10.4^d^		35.7 ± 15.4^d^		33.9 ± 13.6^d^	
	38.4 ± 12.7	36.6 ± 12.8	38.4 ± 9.3	40.3 ± 11.4	39.9 ± 17.0	31.1 ± 12.2^e^	33.6 ± 12.0	34.2 ± 9
SOFA score at admission	8.3 ± 2.5		9.1 ± 1.7^a^		7.5 ± 3.1^a^		6.3 ± 2.9^a^	
	8.2 ± 2.8	8.3 ± 2.2	8.8 ± 1.7	9.3 ± 1.6	7.8 ± 3.5	7.1 ± 3.2	5.7 ± 3.8	6.9 ± 1.4

^a^*P *< 0.0001; ^b^*P *= 0.08; ^c^*P *= 0.14; ^d^*P *= 0.07; ^e^*P *= 0.01. Superscripts a and d refer to comparison between diagnostic categories. Superscripts b, c, and e refer to comparison between AOX and placebo (= *P*) groups. SAPS, Simplified Acute Physiology Score; SOFA, sequential organ failure assessment.

**Table 3:** Specific severity indices in the trauma patients on enrollment, according to presence of brain injury

Trauma (n = 66)	AOX	Placebo	*P *value
Number	34	32	
Injury Severity Score, all	30.1 ± 9.8	28.3 ± 10.4	NS
Brain injury (n = 39; 20/19)	31.0 ± 11.0	28.7 ± 11.9	NS
No brain injury (n = 27; 14/13)	28.9 ± 7.9	27.6 ± 8.1	NS
Injury Severity Score brain, in brain-injured	14.5 ± 4.9	10.8 ± 4.5	0. 019
Glasgow Coma Scale score initial, all	10.4 ± 4.9	11.8 ± 4.3	0.16
Brain injury	7.4 ± 4.3	9.7 ± 4.8	0.11
No brain injury	14.6 ± 0.6	14.8 ± 0.8	NS
SAPS II	39.9 ± 17.0	31.1 ± 12.2	0.04
SOFA score, neuro section	1.62 ± 1.7	0.97 ± 1.2	0.085
Brain injury	2.5 ± 1.6	1.38 ± 1.2	0.012
No brain injury	0.2 ± 0.4	0.2 ± 0.6	NS

Data are presented as mean ± standard deviation. *P *values refer to comparison between the antioxidant (AOX) and placebo groups. NS, not significant; SAPS, Simplified Acute Physiology Score; SOFA, sequential organ failure assessment.

### Protocol violations

There were 47 protocol violations (19 in the placebo group and 28 in the AOX group), evenly distributed among the three diagnostic categories (18 in cardiac, 22 in trauma, and 7 in SAH). Among these, 25 were considered nonsignificant as they reinforced the intervention effect (by increasing the doses of AOX), while 22 violations reduced the difference between groups (5 in placebo patients who received 1 or 2 doses of AOX, 17 in the AOX group by deletion of 1 to 3 doses of supplement). All patients were included in the intent-to-treat analysis.

### Outcome variables

#### Kidney function

Pre-existing renal failure was present in 30.5% of patients, being more frequent in the cardiac surgery patients (*P *< 0.001) but in only 9.5% of SAH patients (Table 4). AKI of any grade developed in 66 (33%) patients (30 and 36, or 29% and 37%, respectively, in AOX and placebo groups; *P *= 0.11); it was most frequent in the cardiac patients (*P *< 0.0001) and least frequent in SAH (2 in AOX and 1 in placebo). The more severe grades of renal failure (increases of 50 μmol/L in 32 patients and of 90 μmol/L in 16 patients) did not differ between AOX and placebo groups. Altogether, 7 patients required transient continuous renal replacement therapy (6 in AOX and 1 in placebo; *P *= 0.05), of whom 6 suffered pre-existing chronic renal failure. After censoring for prior chronic renal failure, there was no significant difference between groups. Persistent renal failure was observed in 11 patients (not significant between groups).

**Table 4:** Outcome variables with detail of cardiac and trauma patients

Variable	All		Cardiac surgery		Trauma	
	AOX	Placebo	AOX	Placebo	AOX	Placebo
Number	200		113		66	
Number	102	98	57	56	34	32
Prior renal failure	61 (30.5%)		49 (43.4%)^a^		10 (15.1%)	
	25	36	20	29	6	4
Acute kidney injury	66 (33%)		50 (44.2%)^a^		13 (19.7%)	
	29 (30%)	36 (37%)	21 (37%)	29 (52%)^b^	7	6
ARF increase of 50 μmol/L	32 (16%)		29 (27.5%)^a^		3 (15.2%)	
	15 (15%)	17 (17%	13 (23%	16 (29%)	2	1
ARF increase of 90 μmol/L	16 (8%)		15 (13.3%)^a^		1 (1.5%)	
	7 (7%)	9 (9%)	6 (11%	9 (16%)	1	0
CVVH	7 (3.5%)		6 (5.3%)		1 (1.5%)	
	6	1^c,d^	5	1	1	0
Persistent renal failure	11 (5.5%)		10 (8.8%)		1 (1.5%)	
	4 (4%)	7 (7%)	3 (5%)	7 (13%)^e^	1	0
Ventilator-free days	26.3 ± 5.5		26.6 ± 5.3		25.2 ± 6.3	
	26.1 ± 5.7	26.6 ± 5.2	25.9 ± 6.7	27.3 ± 3.3^e^	25.5 ± 4.6	24.9 ± 7.7
Infections (patients with)	70		30		30	
	36	34	14	16	17	13
Infectious episodes	91		39		39	
	45	46	19	20	21	18
Pneumonia episodes	30		16		14	
	14	16	7	9	7	7
Length of stay, days						
In the ICU	5.6 ± 5.5		5.2 ± 5.1		6.3 ± 6.5	
	5.8 ± 5.4	5.4 ± 5.7	5.8 ± 6.0	4.7 ± 4.0	5.8 ± 4.4	6.8 ± 8.3
In intermediate care	5.0 ± 5.5		3.6 ± 3.5		6.5 ± 7.2	
	4.5 ± 4.9	5.5 ± 6.0	3.3 ± 4.2	3.4 ± 4.1	5.9 ± 7.1	7.1 ± 7.4
In the hospital	24 ± 20		19 ± 13		32 ± 22	
	23 ± 20	26 ± 20	20 ± 14	19 ± 13	26 ± 19	39 ± 24^f^
Deaths						
In the ICU	14 (7%)		11 (9.7%)		2 (3%)	
	8	5	6	5	2	0^g^
In hospital	23 (11.5%)		12 (10.6%)		8 (12.1%)	
	14	9	8	6	6	2^g^
At 3 months	25 (12.5%)		16 (14.2%)		8 (12.1%)	
	14	11	8	8	6	2^g^

Data are presented as number (percentage) and mean ± standard deviation. ^a^*P *< 0.001. ^b^*P *= 0.109. ^c^*P *= 0.05. ^d^See comment in Results section. ^e^*P *= 0.17. ^f^*P *= 0.016. ^g^*P *= 0.16. Superscript a refers to comparison between diagnostic categories. Superscripts b, c, e, f, and g refer to comparison between antioxidant (AOX) and placebo groups. ARF, acute renal failure; CVVH, continuous veno-venous hemofiltration; ICU, intensive care unit.

#### Sequential organ failure assessment scores

**Figure 2:**
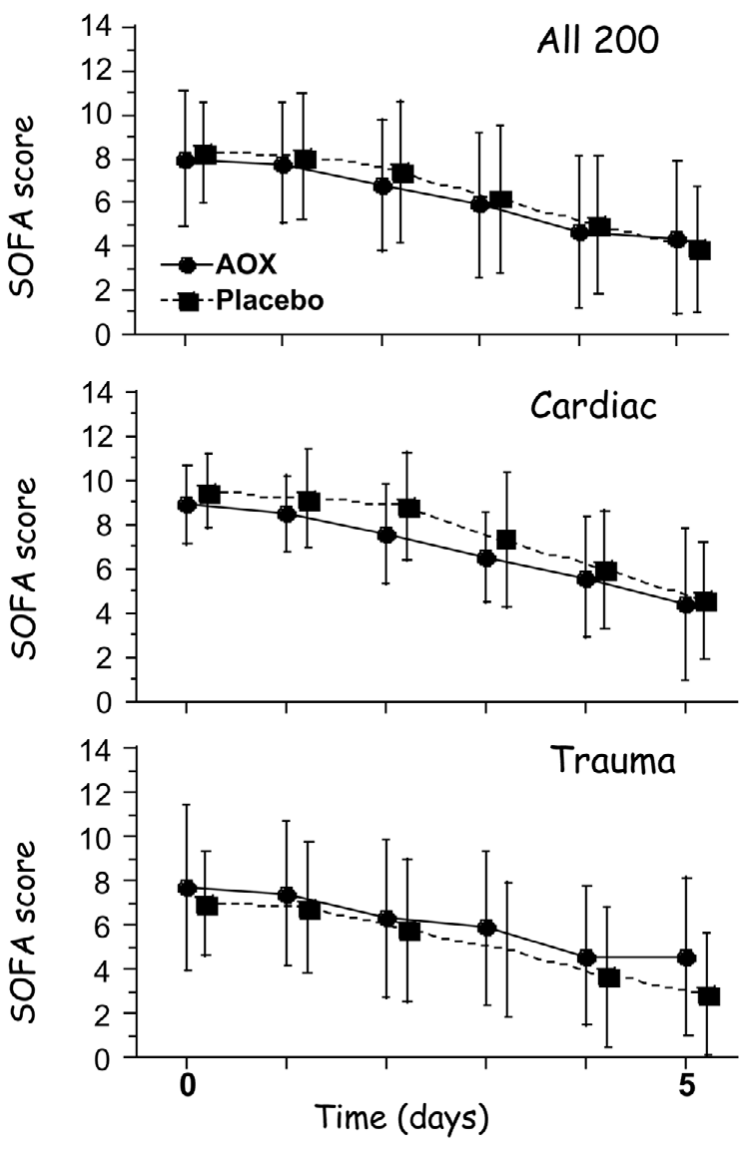
Evolution of the sequential organ failure assessment (SOFA) scores in all patients by group with the detail of cardiac and trauma patients.

Admission scores were elevated but did not differ between treatment groups, although they differed between diagnostic categories (Table 2). The associated initial number of organ failures was also similar (median 3, range 1 to 6), as was the number of severe organ failures (median two organs with a SOFA score of 3 or 4). The SOFA score decreased significantly over time (*P *< 0.0001) in both groups (Figure 2), with no significant difference between AOX and placebo groups.

#### Infections and pneumonia

Seventy patients suffered infectious complications (not significant between groups): the incidence of pneumonia was low (episodes n = 32) and did not differ between groups (Table 4). The likelihood to develop pneumonia increased with energy deficit per kilogram by day 5 (*P *= 0.005) as did that of having any infection (*P *= 0.0015). The lowest infection rate was observed in SAH patients with 13 infections (5 in AOX and 8 in placebo) in 10 patients.

#### Length of mechanical ventilation

Despite lower mean values in the AOX group, the differences did not reach significance (Table 5). The PaO_2_/FiO_2 _ratio increased over time (*P *< 0.0001) in all cardiac and trauma patients, with a trend to faster increase in the AOX patients (data not shown; *P *= 0.109). The number of ventilator-free days did not differ.

**Table 5:** Time course of plasma C-reactive protein (mg/L) in all patients and in the three diagnostic categories from admission to day 5

All	Day 0	Day 1	Day 2	Day 3	Day 4	Day 5
AOX	49 (0–2,350)	129 (2–359)^a^	161 (2–364)^a^	125 (150–399)^a,b^	100 (16–401)^b^	80 (14–401)
Placebo	35 (2–176)	141 (2–341)^a^	174 (2–410)^a^	156 (2–360)^a,b^	114 (2–438)^b^	82 (3–388)
Cardiac surgery						
AOX	59 (2–150)	150 (2–359)^a^	165 (2–317)^a^	123 (20–367)^a^	84 (20–319)^b^	81 (14–243)
Placebo	39 (2–176)	142 (37–341)^a^	177 (53–410)^a^	158 (44–360)^a^	117(26–225)^b^	81 (19–178)
Trauma						
AOX	35 (2–158)	126 (4–282)^a,b^	164 (9–464)^a^	146 (41–399)^a^	146 (41–282)^a^	114 (41–282)^a^
Placebo	32 (2–224)	147 (5–327)^a,b^	201 (46–326)^a^	174 (34–328)^a^	161 (21–438)^a^	117 (34–388)^a^
Subarachnoid hemorrhage						
AOX	32 (0–230)	38 (3–277)	42 (15–184)	67 (16–103)	44 (16–401)	29 (8–401)
Placebo	10 (2–61)	27 (2–185)	22 (2–179)	16 (2–233)	22 (2–233)	21 (2–163)

Data are presented as median (range). C-reactive protein differed over time in the antioxidant (AOX) and placebo groups (two-factor repeated analysis of variance: time effect *P *< 0.0001, group effect *P *= 0.039, and interaction (time*group) *P *= 0.16 to not significant [NS]). The attenuation of inflammation was most significant in cardiac patients (time effect *P *< 0.0001, group effect *P *= 0.41 [NS], and interaction (time*group) *P *= 0.0075) and in trauma patients (time effect *P *< 0.0001, group NS, and time*group NS). No significant change over time was observed in subarachnoid hemorrhage patients. The *post hoc *comparisons were carried out with the Dunnett test (^a^significant difference versus baseline within groups) and the Scheffe test (^b^significant difference between groups at the same time).

#### Length of stay

**Figure 3:**
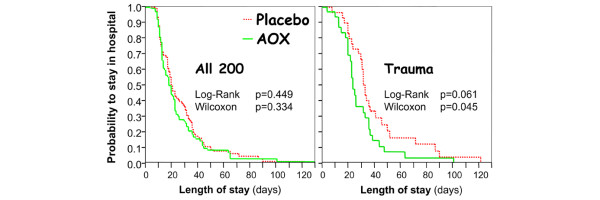
Kaplan-Meier analysis of the length of hospital stay censored for survival according to intervention group in the global population with the detail of trauma patients. AOX, antioxidant.

Stay in the ICU did not differ significantly between groups, although mean lengths of stay were 0.6 and 1.3 days shorter in the AOX group in the surviving cardiac and trauma patients, respectively. The length of hospital stay was 3 days shorter overall in the AOX group, being 13 days shorter in the AOX trauma patients compared with placebo (*P *= 0.016) and 11 days shorter in those AOX trauma patients without brain injury (*P *= 0.053). Gender did not influence outcome. Figure 3 shows that the presence of brain injury was an important determinant of hospital stay.

#### Mortality

Mortality was lower than predicted and did not differ between the groups. While the overall calculated probability of death in the cardiac patients was 15.3% by Euroscore, the observed hospital mortality was 10.6%. In trauma patients, mortality was 12.1%: while mortality between AOX and placebo groups did not differ when all trauma patients were considered (Table 4), the number of deaths tended to be higher in the AOX brain-injured trauma group (*P *= 0.076), with 5 out of 6 deaths directly caused by severe brain injury. Adverse events were collected and did not differ between groups.

### Biological variables

#### Trace elements and glutathione peroxidase

**Figure 4:**
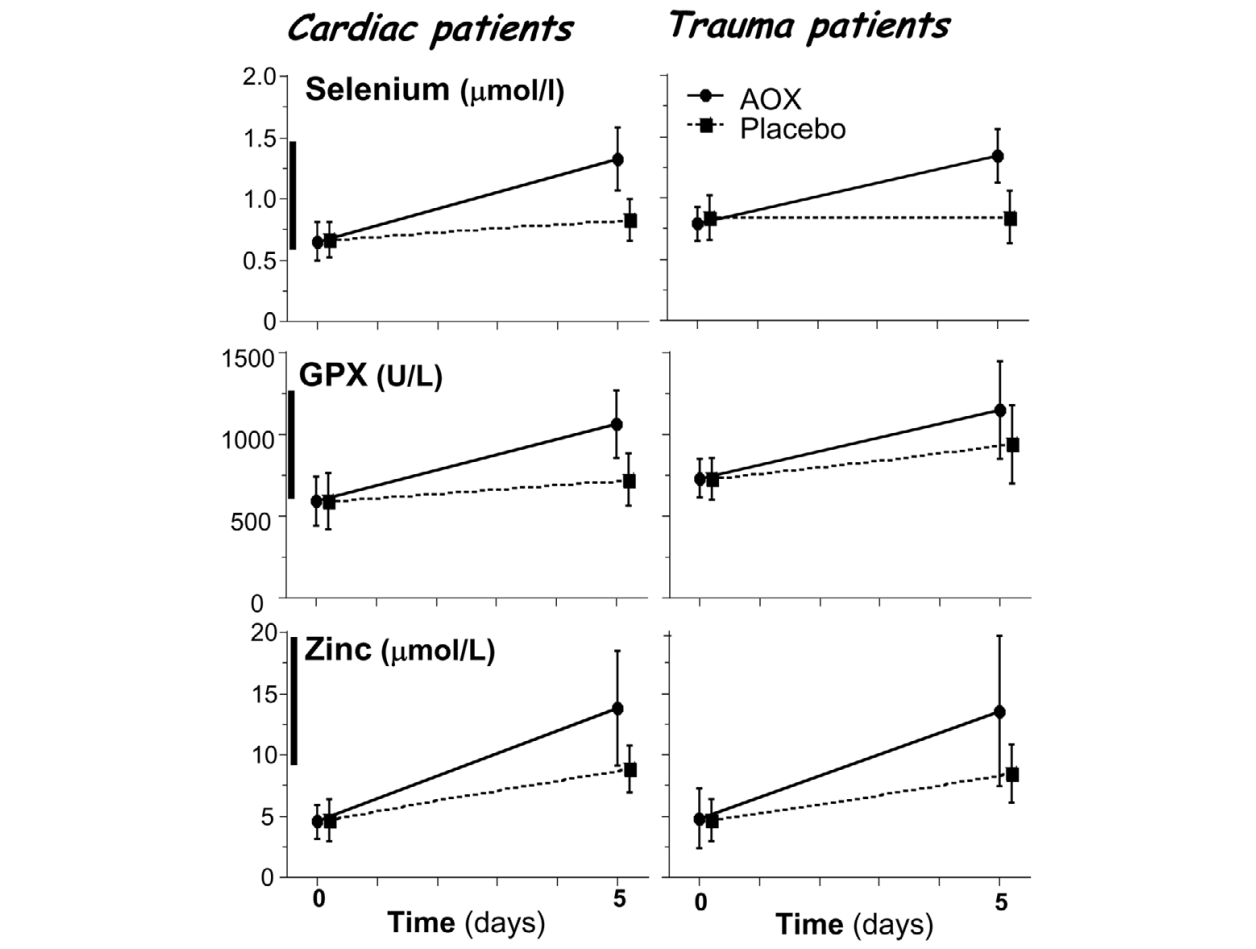
Selenium, glutathione peroxidase (GPX), and zinc plasma concentrations in trauma and cardiac surgery patients. The thick vertical bar next to the y-axis shows the reference ranges. By two-factor repeated measures analysis of variance, the changes over time and the treatment effect (interaction time*group) were strongest in the cardiac group, with *P *< 0.0001 for the three variables, while in the trauma group, treatment effect was selenium *P *< 0.0001, GPX *P *= 0.0013, and zinc *P *= 0.0005. AOX, antioxidant.

Analysis was done in cardiac surgery and trauma patients. Mean selenium, zinc, and GPX concentrations were in the lower normal ranges on admission (not significant between groups) (Figure 4), reflecting typically European conditions, with 69% of selenium and 80% of GPX values being below the lower reference value. The supplements significantly increased plasma concentrations of both selenium and zinc to within the normal ranges. The GPX activity increased with plasma selenium.

#### Plasma C-reactive protein

Mean CRP value peaked by day 2 and decreased in both AOX and placebo groups thereafter (Table 5), and significantly lower values were observed in the AOX patients. The strongest CRP increases were observed after cardiac surgery and in trauma patients, with a faster decay in the AOX cardiac patients. The SAH patients' mean peak and overall CRP values were significantly lower than in the two other categories (*P *= 0.039).

#### Glucose control

The mean blood glucose value did not differ significantly between groups; values from days 0 to 5 were 8.2 ± 2.8, 7.2 ± 1.8, 6.7 ± 1.4, 7.0 ± 1.6, 6.8 ± 1.4, and 7.1 ± 1.8 mmol/L in the AOX group versus 8.9 ± 2.7, 7.4 ± 1.6, 7.2 ± 1.8, 7.1 ± 2.2, 7.1 ± 1.7, and 7.0 ± 1.6 mmol/L in the placebo group. The mean blood glucose over the first 5 days differed by diagnostic category (*P *< 0.0001), being highest in the cardiac surgery (mean of all values = 8.9 mmol/L), intermediate in trauma (8.6 mmol/L), and lowest in SAH (6.2 mmol/L) and decreasing significantly over time (*P *= 0.005), the decrease being similar in the AOX and placebo groups.

#### Insulin requirements

Insulin requirements per day during the first 48 hours were elevated in cardiac surgery patients (not significant between groups) and resulted mainly from the use of glucose-insulin infusions in the cardiac patients, which was dictated by the patients' immediate postoperative condition. The insulin requirements were significantly lower in trauma and SAH patients (*P *< 0.0001), being lowest in the SAH patients, with no difference between groups.

### Nutritional support

Total energy delivery during the first 5 days was hypocaloric in most patients, and the progression of energy delivery was slower than recommended by our protocol. Eleven patients required PN for 3 to 11 days. The mean and median cumulated energy balances on day 5 were negative and did not differ significantly between groups (-5,415 kcal in placebo versus -5,680 kcal in AOX).

### Short Form 36-item health survey

SF-36 could be retrieved in 140 (70%) of the 174 surviving patients (1 cardiac placebo patient died during the fourth month), including 68 AOX and 72 placebo patients (88 cardiac, 36 trauma, and 16 SAH). The 34 missing patients were not feeling well enough to answer (n = 11) or were having language problems (n = 7) or were lost to follow up (altogether, n = 17). Physical activity score (PF) tended to be higher in AOX (24.1 ± 4.9 versus 22.8 ± 5.7; *P *= 0.14). Physical limitation (RP: 5.8 ± 1.4 versus 5.5 ± 1.5; not significant), physical pain (BP: 8.9 ± 2.4 versus 9.0 ± 2.7; not significant), and perceived health (GH: 18.9 ± 4.5 versus 19.2 ± 4.1; not significant) did not differ. Perceived evolution of health after the hospital discharge (HT = Health Transition) was significantly better in the AOX patients, with significantly more frequent ratings 'better' and 'rather better' (*P *= 0.01).

## Discussion

The main result of the present trial is that AOX micronutrient supplements provided for 5 days to critically ill patients did not achieve any significant impact on organ function (acute kidney failure or SOFA score of the first 5 days) despite trends to less renal injury and residual persistent renal failure in the AOX group. The intervention was associated with a significant blunting of the inflammation, reflected by lower CRP levels in the AOX group. Significant reduction of hospital stay was observed only in the trauma group. There was no impact on mortality.

The three diagnostic categories of patients were selected based on the demonstration of the involvement of oxidative stress in their clinical course [17,18], but during the study the three categories behaved differently regarding the systemic inflammatory response. We indeed observed significant differences in the magnitude of the CRP response between the three categories; the complicated cardiac and trauma patients exhibited an intense inflammation, whereas the SAH caused only a limited plasma CRP response, as observed by others [36]. This difference in the inflammatory pattern enables us to generate some hypotheses: (a) in the presence of an intense SIRS, such as in trauma patients, the AOX cocktail downregulated the inflammatory response with clinically observable effects, while (b) there was no detectable biological or clinical effect in those patients with limited SIRS. Considering that SIRS is deleterious since it promotes organ failure [37], such a modulation is potentially beneficial. Indeed, trauma patients appeared to benefit significantly as reflected by the reduction of their hospital stay, which was associated with better perceived health according to the SF-36 score at 3 months. This was particularly true in non-brain-injured patients; in those with brain injury, the conclusion is more awkward due to the higher severity of injury on admission in the AOX group. The improvement of outcome is also in agreement with the reduction of organ alterations observed by Nathens and colleagues [38] in a large vitamin intervention trial in trauma patients. Animal data show that, during the early phase after a brain trauma, the oxidative stress is associated with vitamin E and selenium depletion [39], suggesting a time window for supplementation. In the brain-injured patients, another important question is whether the micronutrients pass the blood-brain barrier and penetrate the injured area. A microdialysis investigation would be required to address this question.

A nutritional intervention requires time to achieve an effect, as shown by prior studies [40]. Nevertheless, positive effects were indeed observed already during the ICU stay and for the 120-day follow-up: CRP levels decreased faster (significantly in the cardiac patients) and several other indicators of outcome had strong trends toward beneficial effects, such as lengths of mechanical ventilation and of ICU stay. In such sick patients, who were particularly unwell, this is a considerable achievement with such a benign and cheap intervention (about $50 USD per day). The presumed rationale for this is a reinforcement of the endogenous AOX defense, as shown by the normalized GPX activity in the treatment group.

### Rationale for the micronutrient doses and combinations

Selenium may be the cornerstone of the AOX defense system in acute conditions [13], but other trace elements, and zinc particularly, are also important players [16]. In our study, the tested selenium dose (540 μg followed by 270 μg/day) can be considered low compared with other recent trials [15,41,42]. Nevertheless, it is a very substantial intake compared with the normal healthy subject requirements of 60 μg per day, and indeed it did correct the plasma selenium concentrations and normalize GPX activity. In the ICU, doses ranging between 350 and 1,000 μg for 10 to 15 days have been associated with clinical benefits [13], while chronic doses of greater than 450 μg/day in the general population have been associated with a reduction of the activity of the 5' triiodothyronine deiodinase (an indicator of upper safe intakes [43]). The reduction in renal failure in inflammatory patients from the first Angstwurm trial was achieved with doses ranging between 100 and 530 μg/day [34] but was not confirmed in subsequent studies with the same dose [44] or higher doses [15]. The latest trial of Forceville and colleagues [42] tested a loading dose of 4 mg followed by 1 mg/day for 2 weeks in 60 septic patients: it did not show any reduction in renal failure, while length of mechanical ventilation was nonsignificantly prolonged (14 days in the placebo group versus 19 days in the selenium group), possibly reflecting an incipient selenium toxicity.

On the other hand, the doses of zinc (2 days of 50 mg then 25 mg/day) and vitamins C, E, and B_1 _were high compared with other trials. This combination was motivated by the inclusion of trauma patients, who develop early negative micronutrient balances and low plasma zinc levels. Furthermore, as neurological damage was present in two of our diagnostic groups, zinc was given based on trials in brain-injured patients in whom 30 to 40 mg zinc doses were associated with improved neurological outcome [45]. Finally, zinc is involved in tissue repair, and the improved wound healing in burns observed with 30 mg iv for 21 days [46] encouraged the addition of this rather large dose of zinc. Indeed, plasma levels normalized with the supplements and trauma patients appeared to benefit from the intervention.

Ascorbic acid, alpha-tocopherol, and thiamine were combined to reinforce the AOX cascade [16]. Vitamin C is strongly depressed in critical illness, and doses of 3 g per day are required to normalize plasma concentrations [47]. A loading dose of 2.7 g for 2 days was therefore adopted. Although chronic administration to healthy subjects of vitamin E of doses exceeding 150 mg/day is associated with increased mortality [48], several trials have delivered 3 g per day for shorter periods to trauma patients and other critically ill patients without any detectable side effects [38,49]. Since the normal daily intake is of the order of 10 mg, our 300 to 600 mg/day dose can be considered at the low range of the high-dose spectrum. Finally, vitamin B_1_, though not an AOX but the coenzyme of all carbohydrate decarboxylation reactions, was delivered to all patients as deficiency is a recognized problem in early critical illness [50], with recommendations to deliver 100 mg and up to 300 mg/day.

### Study limitations

The supplementation did not achieve a significant reduction of the primary outcome (that is, early organ failure). There are different possible explanations. First, we did not collect the SOFA scores after discharge from the ICU – this might have been valuable. Indeed, the impact of a nutritional intervention may be detected only after 4 to 5 days. Then, despite the inclusion of 200 patients in three diagnostic categories, the study was still underpowered. The subgroup analysis is therefore confronted with an even poorer lack of power. Despite this problem, all the mean values were oriented in the direction of a clinical benefit, with trends in favor of the AOX intervention. These trends nevertheless can be considered only as hypothesis-generating: for example, the trauma patients might benefit from such an intervention but a larger trial is required. There was an *a priori *decision to analyze all patients and then by diagnostic category as the constitution of these categories was part of the randomization process. The small SAH group experienced very few complications and had less inflammation in agreement with other studies [36], contributing to the reduction of power. This was worsened by the lower-than-predicted mortality by either of the outcome scores [25]. Furthermore, despite stratification and randomization, we observed heterogeneity among the trauma patients. The more severe brain injuries in the AOX group, which directly impact on survival, explain the trend to higher mortality in this group and must be considered in the analysis [51]. This type of bias is unavoidable, even with strict randomization [35]. Balanced minimization is an efficient method of ensuring comparable groups [52]. It consists of determining, at the onset, outcome factors one would like to see evenly distributed in the groups. The person in charge of the randomization observes the progression of these factors during the trial, modifying group allocation if a difference develops. This method was not used in this study but probably would have avoided this unlucky uneven distribution of severity factors. Finally the characteristic of being a monocentric trial can be considered both a limitation but also an advantage due to reduction of variability of care.

### Conclusion

Selenium-containing AOX micronutrient supplements did not significantly improve organ dysfunction as assessed by the SOFA score during the first 5 days. Nevertheless, the inflammatory response was significantly blunted as shown by a lower CRP in the AOX group. The diagnostic categories with an intense inflammatory response, such as patients with organ failure after cardiac surgery or major trauma patients, thus appear good candidates for an AOX intervention, while patients with limited inflammatory response are not. The optimal dose and combination of micronutrients still remain to be determined but should include selenium and zinc.

## Key messages

• This study confirms that the antioxidant micronutrient status is altered on admission in critically ill patients with organ failure.

• Antioxidant supplements delivered intravenously for 5 days correct the initial alterations and restore antioxidant defenses, particularly the glutathione peroxidase activity.

• There is no impact on organ failures (sequential organ failure assessment) during the first 5 days of admission, but a shorter hospital stay in trauma patients.

• The supplements achieve blunting of the inflammatory response in diagnostic categories with severe systemic inflammatory response syndrome.

## Abbreviations

AKI = acute kidney injury; ANOVA = analysis of variance; AOX = antioxidant; BP = bodily pain; CI = confidence interval; CRP = C-reactive protein; EN = enteral nutrition; FiO_2 _= fraction of inspired oxygen; GCS = Glasgow Coma Scale; GH = general health perception; GPX = glutathione peroxidase; ICU = intensive care unit; ISS = Injury Severity Score; iv = intravenously; MOS SF-36 = Medical Outcome Study Short Form 36-item health survey; PaO_2 _= arterial partial pressure of oxygen; PF = physical functioning; PN = parenteral nutrition; RP = role functioning-physical; RR = relative risk; SAH = subarachnoid hemorrhage; SAPS = Simplified Acute Physiology Score; SF-36 = Short Form 36-item health survey; SIRS = systemic inflammatory response syndrome; SOFA = sequential organ failure assessment.

## Competing interests

The study was supported by a grant from Fresenius Kabi AG (Bad Homburg, Germany) to the department that partly financed the salary of LS (no direct payment) and provision of the study micronutrients. MMB, AS, J-PR, and RLC have given conferences and lectures supported by the same company. AS and RLC have also delivered conferences sponsored by Nestlé (Vevey, Switzerland) and by B. Braun (Melsungen, Germany). There are no patents under development. There are no other conflicts of interest (financial or nonfinancial) to disclose.

## Authors' contributions

MMB contributed to the conception and design of the study, clinical investigation, data collection, data analysis, and manuscript preparation. LS contributed to the data collection, data analysis, and manuscript preparation. AS contributed to the conception and design of the study, development of analytical methods, data analysis, and manuscript preparation. J-PR contributed to the conception and design of the study, clinical investigation, interpretation of data, data analysis, and manuscript preparation. CP contributed to the data analysis and manuscript preparation. MB contributed to the development of analytical methods, interpretation of data, and manuscript preparation. RLC contributed to the conception and design of the study, data analysis, and manuscript preparation. All authors read and approved the final manuscript.
